# Draft genome of an anaerobic nitrate-reducing, benzene-degrading member of the order *Thermincolales*

**DOI:** 10.1128/mra.00295-24

**Published:** 2024-08-27

**Authors:** Johnny Z. Xiao, Camilla L. Nesbø, Olivia Molenda, Courtney R. A. Toth, Elizabeth A. Edwards

**Affiliations:** 1 Department of Chemical Engineering and Applied Chemistry, University of Toronto, Toronto, Ontario, Canada; 2 Department of Biological Sciences, University of Alberta, Edmonton, Alberta, Canada; California State University San Marcos, San Marcos, California, USA

**Keywords:** anaerobic, benzene, nitrate reduction, metagenome assembled genome, *Thermincolales*, carboxylase

## Abstract

We present a metagenome assembled genome (MAG) of an anaerobic bacterium from a nitrate-reducing, benzene-degrading enrichment culture (NRBC). The draft *Thermincolales* genome consists of 20 contigs with a total length of 4.09 Mbp and includes putative carboxylase genes likely involved in benzene activation.

## ANNOUNCEMENT

NRBC [previously known as Cartwright-NO_3_ (1)] is a nitrate-reducing, benzene-degrading microbial enrichment culture established in 1995 from a gasoline-contaminated site (*Latitude*: 43.722647, *Longitude*: −79.463022), and is grown in a defined anaerobic mineral medium repeatedly amended with benzene (300–400 µM) and ~2 mM nitrate (2, 3). A benzene-degrading bacterium first classified as a *Peptococacceae* by 16S rRNA gene sequence analysis (NCBI accession: KJ522755.1) was identified in the mixed culture (1); it was later grouped within the *Thermincola* genus (4) using the SILVA SSU 138 database (5). Here, we report a near-complete genome of this uncultured benzene-degrading bacterium to better understand its phylogeny and metabolism.

The assembly of the *Thermincola* MAG used multiple previously reported data sets (6). Illumina paired-end (NCBI accession: SRR24043423) and mate-pair (NCBI accession: SRR24043417) reads were obtained in 2013 from an NRBC subculture called CartCons19. Paired-end reads were sequenced using the TrueSeq DNA Library Prep kit LT, and mate-pair reads were sequenced using the Nextera Mate Pair Library Preparation Kit, according to the manufacturer’s (Illumina) instructions with no additional quality assurance measures. All raw reads were processed using Trimmomatic v. 0.32 (7) before using Abyss v. 1.3.7 (8) to create unitigs that were merged with scaffolds generated in ALL-PATHS-LG v. 4.7.0 (9) using a gap-filling Perl script (10) based on the script in Text S1 of Tang et al. (11). Due to the high number of undefined nucleotides in this metagenome assembly (JARXNP010000000), further steps were taken. In 2018, a NRBC subculture (FeS-Dialysis) was sequenced using the HiSeq PE Cluster Kit v4 cBot (Illumina) with no additional quality control measures (NCBI accession: SRR24043422) and was assembled using IDBA v. 1.1.1 (12) and binned using metaBAT v. 2.12 (13). A bin assigned to *Thermincola* in 157 contigs (NCBI accession: JAVSMV000000000.1) was retrieved as reported previously (6). These 157 contigs were incorporated into the above Abyss/ALL-PATHS-LG gap-filling workflow generating a 26-contig assembly that was curated by read mapping using BBMap v. 38.94 (14) to resolve ambiguities. Finally, long reads from a 2020 NRBC subculture called 10L-NRBC, sequenced according to the manufacturer’s instructions using PacBio RSII with the SMRTbell Express Template Prep Kit 2.0 (SRR24043419) without shearing or size selection (Pacific Biosciences), were used to join adjacent contigs using the *de novo* assembly tool in Geneious v. 8.1.8 (15), resulting in a 20-contig assembly. Read mapping visualization was done using Geneious v. 8.1.8, and genome annotation was performed using NCBI’s Prokaryotic Genomic Annotation Pipeline, PGAP v. 1.2 (16). A more detailed description with all data sets and scripts is provided in Figshare (10).

**Fig 1:**
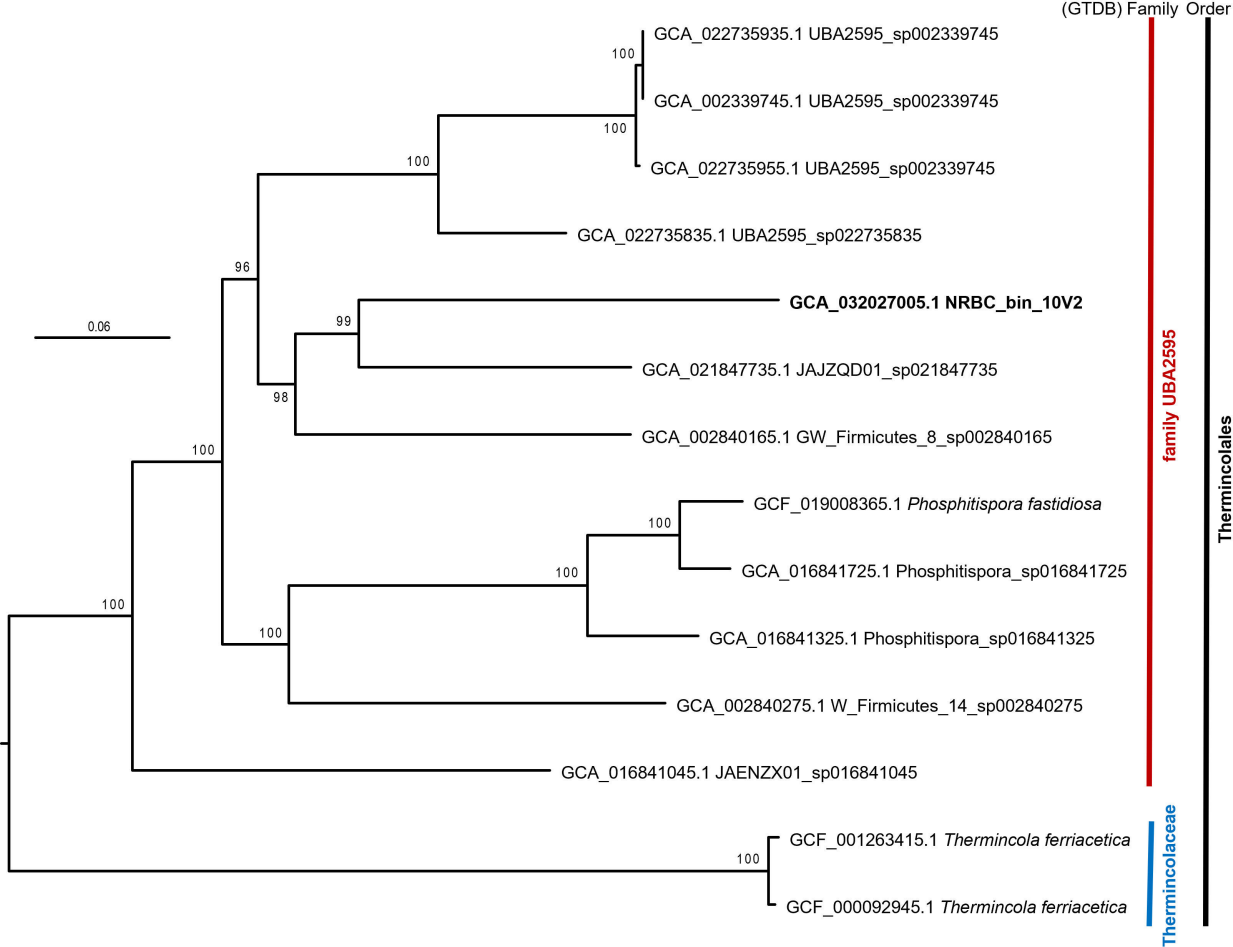
Maximum likelihood phylogenetic tree of *Thermincolales* genomes. The MAG reported is highlighted in bold. The remaining genomes are other members from the order *Thermincolales* from GTDB R214.1 obtained using the GToTree v1.8.1 pipline (20). Single-copy conserved genes were extracted using the Firmicutes.hmm profile and aligned by MUSCLE and concatenated by GToTree. The 23,147 amino acid alignments were imported to Geneious Prime v2023.2.1 (15), sites with >90% gaps were removed, and a maximum likelihood tree was constructed using RAxML v8 (21) with the WAG+GAMMA substitution model and 100 bootstrap replicates. Bootstrap support values are reported as numbers on branches. The tree was rooted by midpoint rooting. The GTDB order and family assignments are shown on the right.

The 20-contig MAG is 4,085,792 bp with an average GC content of 44.5%, N_50_ of 277,970 bp, 100% completeness, and 0% contamination scores, as determined by CheckM v. 1.2.2 using the lineage-wf command (17). At least four distinct 16S rRNA amplicon sequence variants classified to the genus *Thermincola* have been identified in previous sequencing analyses of NRBC subcultures (4); however, no complete 16S rRNA genes were incorporated into the 20-contig MAG. Phylogenomic analysis using GTDB-tk (Release 214.1) classifies this MAG at the order level as a member of the *Thermincolales* (18, 19) in the placeholder family UBA2595 and likely represents a novel genus within the *Thermincolales* (Fig. 1).

## Data Availability

The 20-contig version of the *Thermincolales* MAG has been deposited to NCBI under the accession number JAVSMW000000000.1. The original 157-contig MAG was previously deposited under accession number JAVSMV000000000.1. Illumina reads can be found under accession numbers SRR24043417, SRR24043423, and SRR24043422, whereas PacBio reads are found under accession number SRR24043419. Assemblies and bins are available at NCBI under Project PRJNA951427 and on FigShare (https://doi.org/10.6084/m9.figshare.22637596.v4). The latter also includes MAG statistics and FASTA files as well as detailed assembly steps and scripts. An older version of this draft genome is available in the US DOE Joint Genome Institute’s Integrated Microbial Genomes (IMG) system (ID: 2835707023).
